# Disastrous cerebral and ocular vascular complications after cosmetic facial filler injections: a retrospective case series study

**DOI:** 10.1038/s41598-024-54202-w

**Published:** 2024-02-12

**Authors:** Fanfan Zhao, Yao Chen, Dong He, Xiangxi You, Yuyun Xu

**Affiliations:** 1 Center for Rehabilitation Medicine, Department of Radiology, Zhejiang Provincial People’s Hospital (Affiliated People’s Hospital), Hangzhou Medical College, Hangzhou, Zhejiang China; 2 Department of Radiology, 903 Hospital of the Joint Service Support Force of the Chinese People’s Liberation Army, Hangzhou, Zhejiang China; 3 Department of Radiology, The First People’s Hospital of Jiashan, Jiaxing, Zhejiang China

**Keywords:** Anatomy, Diseases, Health care, Pathogenesis, Risk factors

## Abstract

Soft tissue filler injections are among the most popular facial rejuvenation methods. Cerebral infarction and ophthalmic artery occlusion are rare and catastrophic complications, especially when facial cosmetic fillers are injected by inexperienced doctors. Radiologists and plastic surgeons need to increase their awareness of the complications associated with fillers, which allows early diagnosis and intervention to improve patient prognosis. Regarding the mechanism by which vascular occlusion occurs after facial filler injections, a retrograde embolic mechanism is currently the predominant theory. Numerous case reports have been presented regarding complications associated with injections of facial aesthetics. However, the small sample sizes of these studies did not allow for an adequate assessment of the clinical and imaging manifestations based on the location of the occlusion and the type of filler, and detailed elaboration of multiple cerebral infarctions is also lacking. Therefore, this study aimed to investigate the clinical and radiological features of severe cerebral and ocular complications caused by cosmetic facial filler injections. In addition, we discuss the pathogenesis, treatment, and prognosis of these patients. The clinical, computed tomography (CT), magnetic resonance imaging (MRI), and digital subtraction angiography (DSA) findings were described and analysed. Radiological examinations are crucial for demonstrating severe complications, and brain MRI is especially strongly suggested for patients with cosmetic filler-induced vision loss to identify asymptomatic cerebral infarctions. Extreme caution and care should be taken during facial injections by plastic surgeons.

## Introduction

Soft tissue filler injections are among the most popular nonsurgical facial rejuvenation methods worldwide; more than 5.5 million filler injections were used in 2014, and more than $11 billion in revenue was generated annually^1,2^. Among these fillers, hyaluronic acid (HA) is a frequently used injectable filler, and more than 800,000 Americans receive HA injections each year due to its durability, biocompatibility, reabsorption, and cost effectiveness^3^. Despite the high safety profile of HA, complications can occur, especially when HA is injected by inexperienced doctors or via substandard "syringes"^4^.

**Figure 1:**
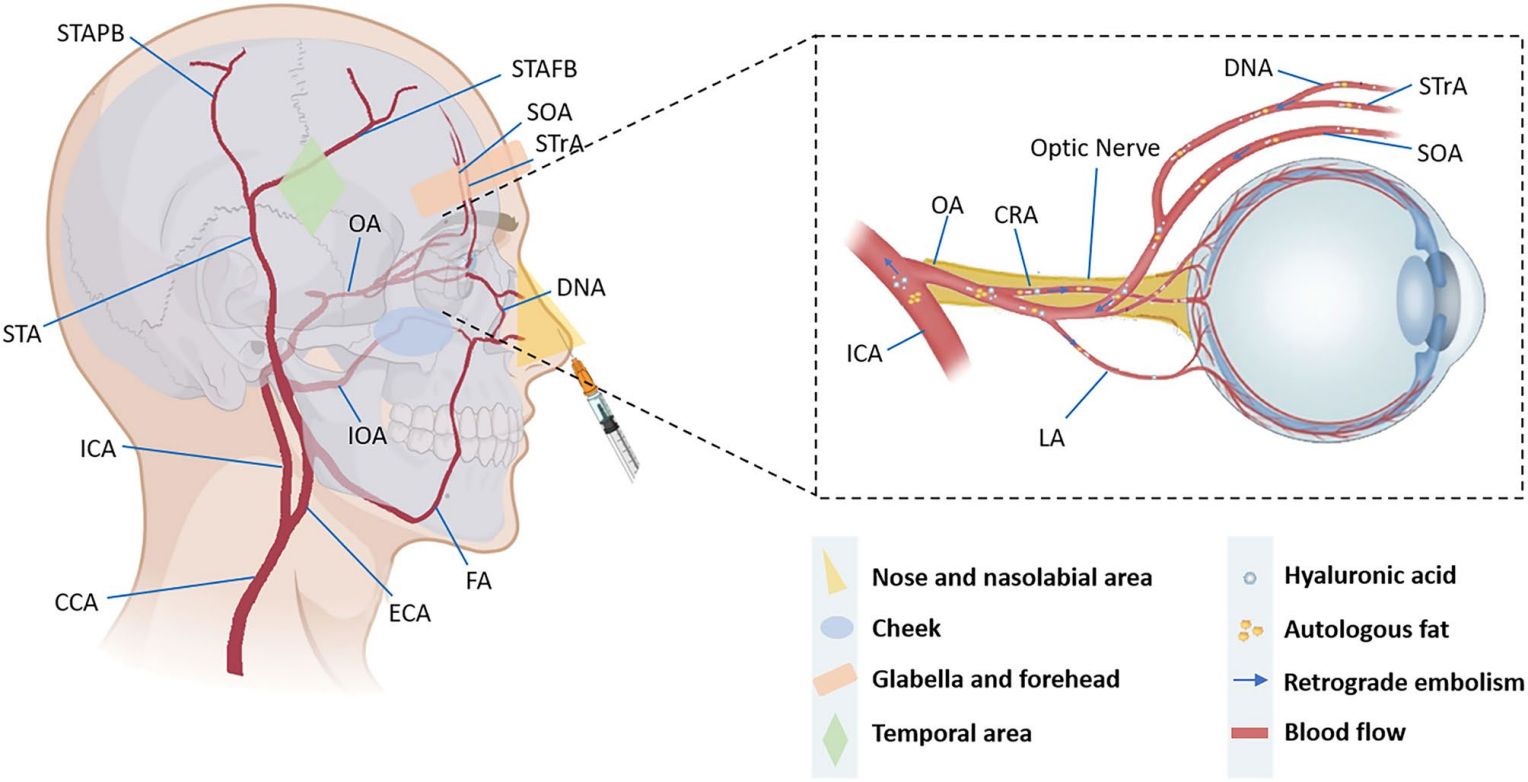
Schematic drawing of the facial region vascular anatomy and the possible obstruction mechanism of hyaluronic acid and autologous fat injection. The illustration shows that the substances in the injection region flowed back into the internal carotid artery and small facial arteries due to pressure and the vascular network, resulting in complications such as ocular and cerebral infarction, skin ischaemia and necrosis. CCA, Common carotid artery; ICA, internal carotid artery; ECA, external carotid artery; IOA, infraorbital artery; FA, facial artery; STA, superficial temporal artery; STrA, supra-trochlear artery; SOA, supraorbital artery; STAFB, superficial temporal artery frontal branch; STAPB, superficial temporal artery parietal branch; DNA, dorsal nasal artery; OA, ophthalmic artery; CRA, central retinal artery; LA, lacrimal artery.

Cerebral infarction and ophthalmic artery occlusion are rare but catastrophic complications of cosmetic filler injections^5^; they occur mainly after filler injection into the glabellar and nasal regions, the nasolabial fold, or the forehead, in order of reported cases, with a rather low overall incidence^6^. The mechanism of retinal artery occlusion after facial cosmetic filler injection is proposed to be retrograde embolization. The backflow of substances from the injection area into the internal carotid artery and small facial arteries occurs due to pressure and the vascular network, leading to complications such as ocular and cerebral infarction, skin ischaemia and necrosis^7^ (Fig. 1). Patients typically present with sudden vision loss, headache, altered consciousness, and limb weakness during or shortly after the filling procedure. Rapid and extensive cerebral ischaemia and postinfarction haemorrhage can lead to irreversible brain damage and even death^4^.

Therefore, awareness of serious filler-related complications is crucial, as patient outcomes could be improved with early diagnosis and appropriate interventions^8^. Attempts towards determining the radiological manifestations of complications resulting from HA filler injection have already been reported. Kim et al.^9^ proposed cerebral angiographic features of ophthalmic and retinal artery obstruction associated with cosmetic facial fillers in seven patients undergoing intra-arterial thrombolytic therapy (IATT). However, due to the small sample size, clinical and imaging features could not be adequately assessed on the basis of the location of the occlusion or the type of filler. Park et al.^10^ conducted a nationwide survey to describe the clinical and angiographic features of 44 Korean cases with medically induced occlusion of the ophthalmic artery and its branches due to cosmetic facial filler injections. This study generally presents the imaging manifestations of complications following facial filler injections and classifies occlusions of the ophthalmic artery and its branches into 6 types according to fluorescein angiographic findings. However, one limitation of this approach is the lack of detailed descriptions of cerebral infarctions caused by these injections.

This study aimed to investigate the clinical and radiologic features of iatrogenic occlusion of the cervical-cerebral artery and its branches caused by cosmetic facial filler injections. In addition, we discuss the pathogenesis, treatment, and prognosis of these patients and briefly review the related literature.

## Methods

### Study sample

The Ethics Committee of Zhejiang Provincial People's Hospital (ZJPPH) review board approved this retrospective study and waived the requirement for written informed consent. To protect patient privacy, all the data were desensitized before use, and relevant prescribed guidelines were implemented in this study.

**Figure 2:**
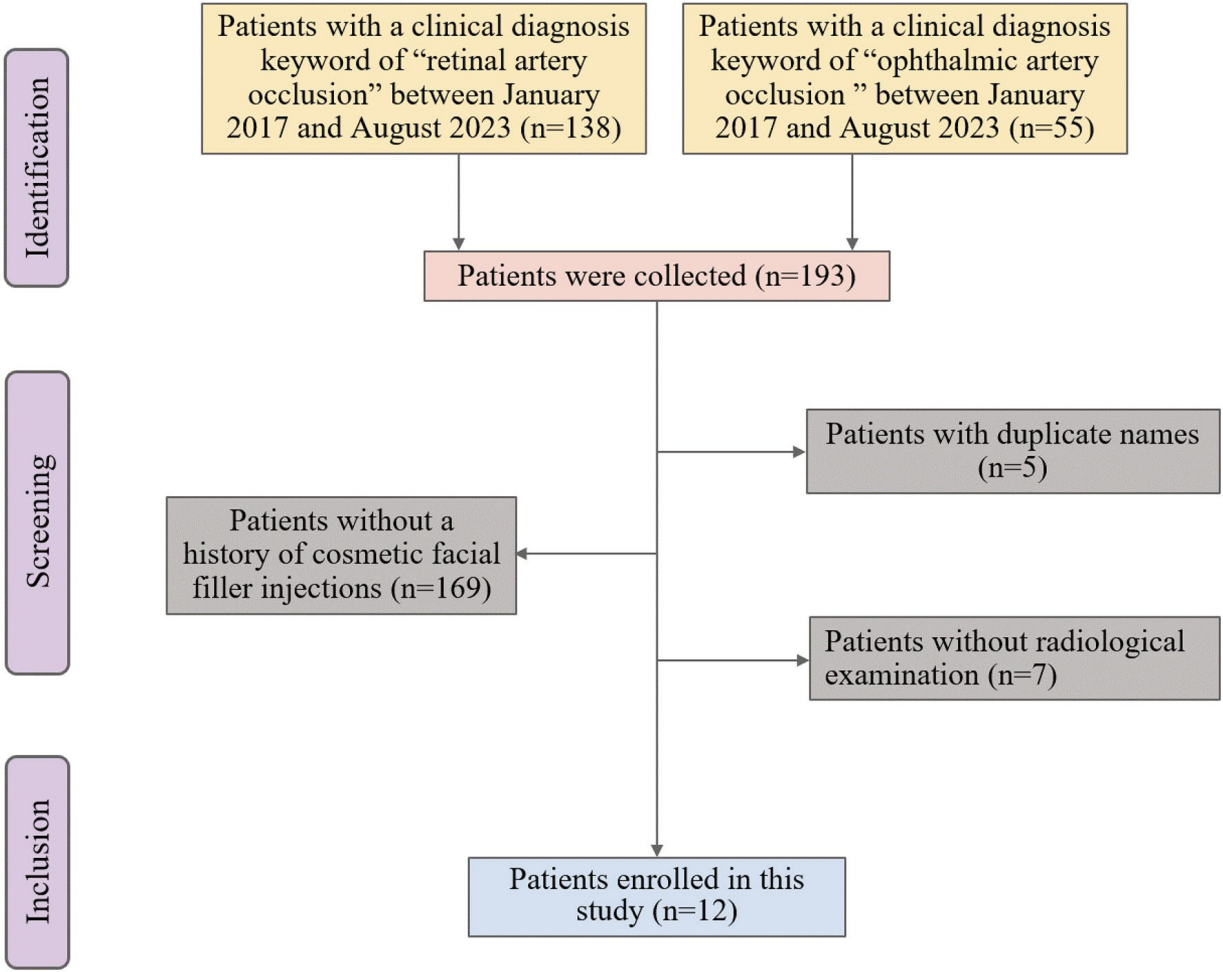
Flow diagram of the process used to select subjects for this study.

Between January 2017 and August 2023, a total of 193 patients with a clinical diagnosis of “retinal artery occlusion” or “ophthalmic artery occlusion” were collected from the ZJPPH. Some patients were excluded from this study to ensure that the complete radiological manifestations were observed. The exclusion criteria were as follows: patients with duplicate names (n = 5), patients without a history of cosmetic facial filler injections (n = 169), and patients without radiological examination (n = 7). Finally, twelve patients were included in this study (Fig. 2).

We reviewed all the patient demographics, clinical characteristics, and imaging manifestations from the electronic medical records, including the injection filler material, injection site, injection dose, time interval between the injection and symptoms, initial and final visual acuity, initial symptoms and signs, diagnosis, treatment, hyaluronidase quantity, brain computed tomography (CT), magnetic resonance imaging (MRI), and digital subtraction angiography (DSA)^11^.

The cases of iatrogenic retinal artery occlusion were classified on the basis of the presumed location obtained from fundus photographs and angiographic findings as follows^10^: (1) ophthalmic artery occlusion (OAO), (2) central retinal artery occlusion (CRAO), (3) branch retinal artery occlusion (BRAO), (4) supraorbital artery (SOA), and (5) ischaemic optic neuropathy (ION).

### Imaging acquisition

CT: Unenhanced head CT scans were performed at a 5-mm slice thickness for six patients. The following scanner settings were used: 100–120 kVp, 512 × 512 matrix, and automatic tube current. CT angiogram (CTA) and CT perfusion (CTP) on an Aquilion One system (Toshiba Medical Systems, Otawara, Japan). Intravenous injection of 50 mL of Iohexol containing 350 mg of iodine per mL (Ominipaque, GE Healthcare, China) was administered at an injection rate of 3–4 mL/s. CT perfusion images were subsequently transmitted to the postprocessing platform to obtain cerebral blood volume (CBV), cerebral blood flow (CBF), mean transit time (MTT), time to peak (TTP), and delay time images.

MRI: Clinical routine MR images were obtained with a 3.0 T MRI scanner (Discovery MR 750, GE Healthcare) with an eight-channel head coil using the same MR parameters for all patients, including axial T1-weighted imaging (T1WI), T2-weighted imaging (T2WI), diffusion-weighted imaging (DWI), and T2-weighted fluid-attenuated inversion recovery (T2-FLAIR) images. All the sequences were performed with a section thickness = 5.5 mm and an interslice gap = 1.5 mm. Susceptibility-weighted imaging (SWI) was performed for only one patient to investigate intracranial haemorrhage.

DSA: Cerebral DSA was performed on a Philips Medical Systems machine at six fps and 75 kVp. Superselective intra-arterial thrombolysis with hyaluronidase was attempted for four patients after providing informed consent. The patients were placed in the supine position, and after routine local disinfection, a 6F catheter sheath was placed on the C1 level using the Seldinger technique. Then, the microcatheter was introduced along with the guidewire, the tip of the microcatheter was placed at the proximal beginning of the ophthalmic artery, and 1500 U or 1200 U hyaluronidase (in combination with 10 ml of saline) was slowly injected^12^.

### Analysis of radiological data

All radiological data were analysed by two neuroradiologists (F.Z. and Y.C., with 5 and 8 years of work experience, respectively) who independently reviewed the images. Any discrepancy between the two doctors was resolved by consulting with a third board-certified radiologist (Y.X., with 17 years of head and neck radiology work experience). The imaging findings of the lesions, such as the location, size, CT density, signal intensity on MRI, cerebral artery morphology on CTA, and perfusion changes on CTP, were reviewed.

## Results

### Study population

Twelve patients (10 patients who received HA injections and 2 patients who received autologous fat injections) with ophthalmic or retinal artery obstruction associated with facial cosmetic filler injections were analysed in this study. A total of twelve women were included, and the mean age was 38.5 ± 11.3 years (range, 22–61 years). The amount of HA filler injected was 0.1–0.3 ml. The mean time from onset to hospitalization was 19.4 h (range 0.5–72 h), and the mean follow-up period was 25.7 ± 21.3 days (range, 7–90 days).

### Clinical characteristics

The nose area was the most common site at which HA was injected (50%, 5/10), and caused occlusion of the artery (2 patients with OAO, 2 patients with CRAO, 1 patient with BRAO, and 2 patients with ACI). The second most common site was the glabellar region (n = 3, 1 patient with OAO, 1 patient with CRAO, 1 patient with BRAO and SOA), followed by the preorbital region (n = 1, 1 patient with CRAO and ACI) and the cheek area (n = 1, 1 patient with CRAO and ACI). However, the injection of autologous fat entirely into the cheek (100%, 2/2) resulted in CRAO and OAO, respectively. A total of 66.7% (8/12) of patients did not receive IATT after the embolism occurred but were treated with a retrobulbar injection of hyaluronidase (RIH), anterior chamber paracentesis (ACP), or massage at the hospital. A total of 33.3% (4/12) of patients who were provided with an IATT showed improvement in visual acuity. The visual prognosis was poor, with 5 patients (41.7%) having a final visual acuity of no light perception (NLP). The demographic and clinical characteristics are presented in Table 1.

**Table 1:** Demographic and clinical data of the patients.

Case No	Sex/age (y)	Eye	Diagnosis	Cosmetic Injection			Symptom to hospital (h)	Initial symptoms	Treatments	Hyaluronidase	Visual acuity		Follow-up (day)
				Material	Site	Dose (ml)					Initial	Final	
1	F/46	R	CRAO, ACI	HA	Preorbital	0.1	4	SLOV	IATT	1500U	NLP	LP	7
2	F/31	R	OAO, ACI	HA	Nose	–	5	SLOV, headache and left limb paralysis	RIH	–	NLP	NLP	14
3	F/31	L	CRAO, ACI	HA	Nose	0.2	4.5	SLOV, headache, nausea and vomiting,	IATT	1500U	NLP	0.2	24
4	F/31	R	OAO	HA	Glabella	0.3	0.5	SLOV, ocular pain, headache, ptosis	IATT	1500U	NLP	LP	15
5	F/61	R	OAO	HA	Nose	0.2	20	SLOV, ophthalmoplegia	RIH	1500U	0.2	0.3	90
6	F/22	R	CRAO, ACI	HA	Cheek	0.1	8	SLOV, headache	ACP	–	NLP	NLP	16
7	F/38	R	CRAO	HA	Glabella	0.1	4	Visual acuity decrease	RIH	300U	CF	CF	26
8	F/52	R	BRAO	HA	Nose	0.1	48	Visual acuity decrease, ocular pain	IATT	1200U	NLP	LP	23
9	F/43	R	OAO	Fat	Cheek	–	10	SLOV, ocular pain	Massage	–	NLP	NLP	18
10	F/27	L	CRAO	HA	Nose	0.1	72	SLOV, headache, nausea and vomiting	RIH	1000U	NLP	NLP	30
11	F/35	R	BRAO, SOA	HA	Glabella	0.2	9	SLOV, headache, ocular pain, ptosis	RIH	1000U	NLP	CF	29
12	F/46	R	CRAO	Fat	Cheek	–	48	SLOV	ACP	–	NLP	NLP	16

ACI: Acute cerebral infarction; CRAO: central retinal artery occlusion; OAO: ophthalmic artery occlusion; BRAO: branch retinal artery occlusion; SOA: supraorbital artery; HA: hyaluronic acid; SLOV: sudden loss of vision; IATT: intra-arterial thrombolytic therapy; RIH: retrobulbar injection of hyaluronidase; ACP: anterior chamber paracentesis; CF: counting fingers; LP: light perception; NLP: no light perception.

### Radiologic findings

The main imaging findings of the patients are summarized in Table 2. In this study, eight patients underwent MR imaging of the brain, and the results showed abnormalities. Six patients also had corresponding neurologic symptoms, including headache (5 patients), contralateral hemiplegia (2 patients), and urinary incontinence (2 patients). DSA was performed in only 4 patients.

**Table 2:** Radiological manifestations of the patients.

Case No	Neurological symptoms and signs	CT/CTA	MRI	Cerebral infarction	DSA
1	None	Not available	ACI in right frontal and occipital lobes	Multifocal	Right CRAO
2	Headache and left limb paralysis, fatigue,	Head CT of 5 h after onset is normal. CTA of 7.5 h after onset shows right OAO	ACI in bilateral frontal, right parietal, and occipital lobes; Right ION SWI shows haemorrhage lesion two days later	Multifocal	Not performed
3	Headache, and right limb paralysis, urinary incontinence	Head CT scan after 3 h, 10 h, and 18 h of shows normal	ACI in left frontal and parietal lobes, left head of caudate nucleus	Multifocal	Left CRAO, left MCA thromboses
4	Headache, ptosis, nausea and vomiting	Head CT scan after 1 h shows normal	Not available	Not available	Right OAO
5	Headache, Nausea and vomiting, ophthalmoplegia	Not available	Not available	Not available	Right OAO
6	None	CTA of 8 h after onset shows OAO	ACI in bilateral frontal and parietal, left basal ganglia, right occipital lobes SAH; Right ION	Multifocal	Not performed
7	None	Head CT scan after 4 h shows normal	Right ION	Not available	Not performed
8	None	Head CT scan after 2 h shows normal	Not available	Not available	Not performed
9	None	CTA of 7.5 h after onset shows right OAO	Right ION	Not available	Not performed
10	Urinary incontinence	Not available	ACI with haemorrhage in the left frontoparietal lobes, Left ION	Multifocal	Not performed
11	Headache, ptosis	Head CT scan shows normal	Not available	Not available	Not performed
12	None	Not available	Right ION	Not available	Not performed

ACI: Acute cerebral infarction; OAO: ophthalmic artery occlusion; CRAO: central retinal artery occlusion; BRAO: branch retinal artery occlusion; ION: ischemic optic neuropathy; HA: hyaluronic acid.

**Figure 3:**
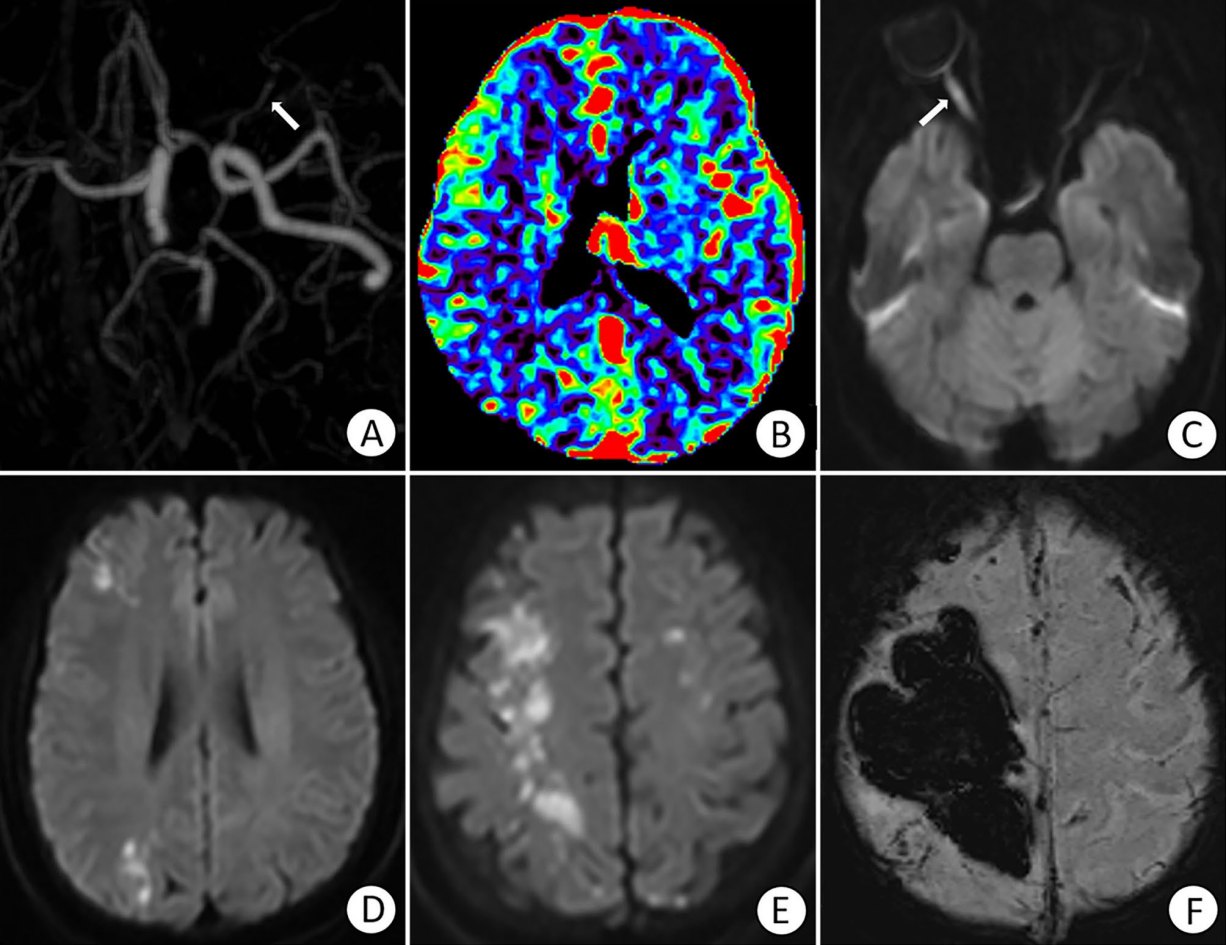
(**A**) CTA image of a patient 7.5 h after filler injection. The white arrow shows the normal left ophthalmic artery, while the right ophthalmic artery cannot be observed, reflecting its occlusion. (**B**) CT perfusion image showing that the time to peak perfusion was normal. (**C**) Diffusion-weighted imaging after 20 h showing a segmental hyperintense lesion in the thickened optic nerve of the right eye. (**D**, **E**) Diffusion-weighted imaging showing watershed infarctions in the bilateral frontal and parietal lobes and right occipital lobe. (**F**) Susceptibility-weighted image obtained after two days indicating postinfarction haemorrhage in the right cerebral hemisphere.

CT images: All eight noncontrast head CT scans from six patients obtained between 1 and 18 h after onset were negative. CTA of Patient 2 (Fig. 3) showed occlusion of the right ophthalmic artery and a normal left ophthalmic artery, while the CT perfusion image of the same patient showed that the perfusion parameters (CBF, CBV, MTT, and delay time) of the brain were normal.

**Figure 4:**
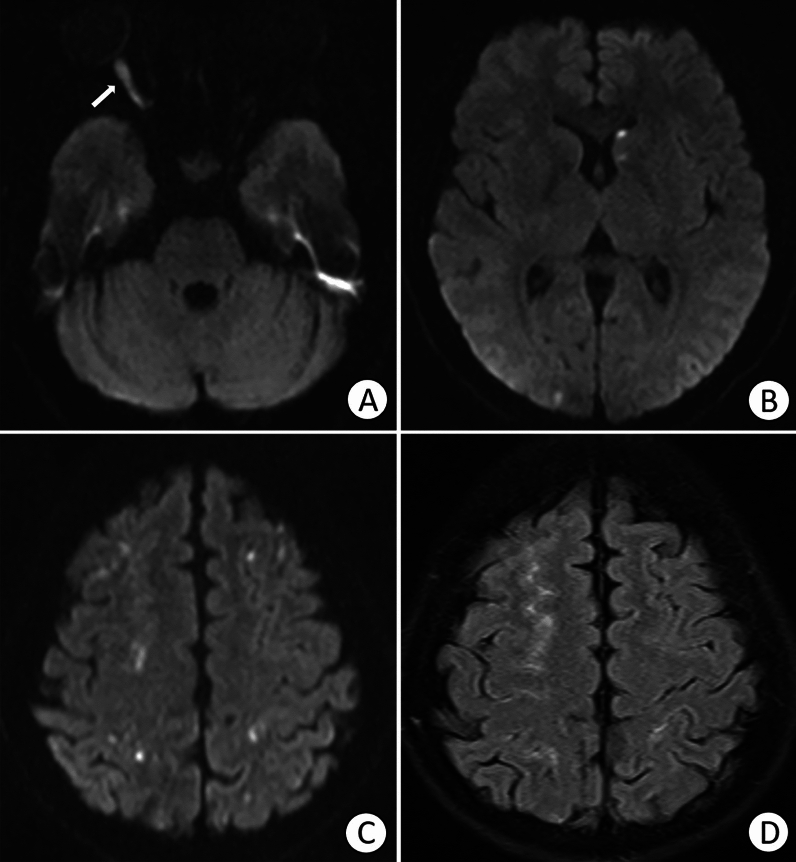
A 22-year-old female patient presented with a sudden loss of vision. (**A**) Diffusion-weighted imaging showing acute right optic nerve ischaemia. (**B**, **C**) Diffusion-weighted imaging showing scattered acute cerebral infarctions, mainly in the bilateral watershed areas. (**D**) Fluid-attenuated inversion recovery image showing subarachnoid haemorrhage.

MRI characteristics: Five of the 12 patients (41.7%) who underwent brain MR imaging exhibited multifocal acute/subacute infarction, representing 62.5% of all patients with a brain lesion and three unilateral and two bilateral cerebral hemispheres, mostly involving the frontal and parietal lobes, especially the watershed zones. The size of the lesions ranged from 3 mm to 7.5 cm in the greatest dimension. All lesions showed hypointense to isointense signals on T1WI and hyperintense to isointense signals on T2WI. Intracerebral haemorrhage was observed on SWI in Patient 2, as shown in Fig. 3, and when the patient’s symptoms worsened, she was transferred to another hospital. Subarachnoid haemorrhage (SAH) was observed on FLAIR in Patient 6. Acute ischaemia of the right optic nerve manifested in Patients 2 and 6, with a swollen optic nerve and diffusion restriction on DWI (Figs. 3 and 4).

**Figure 5:**
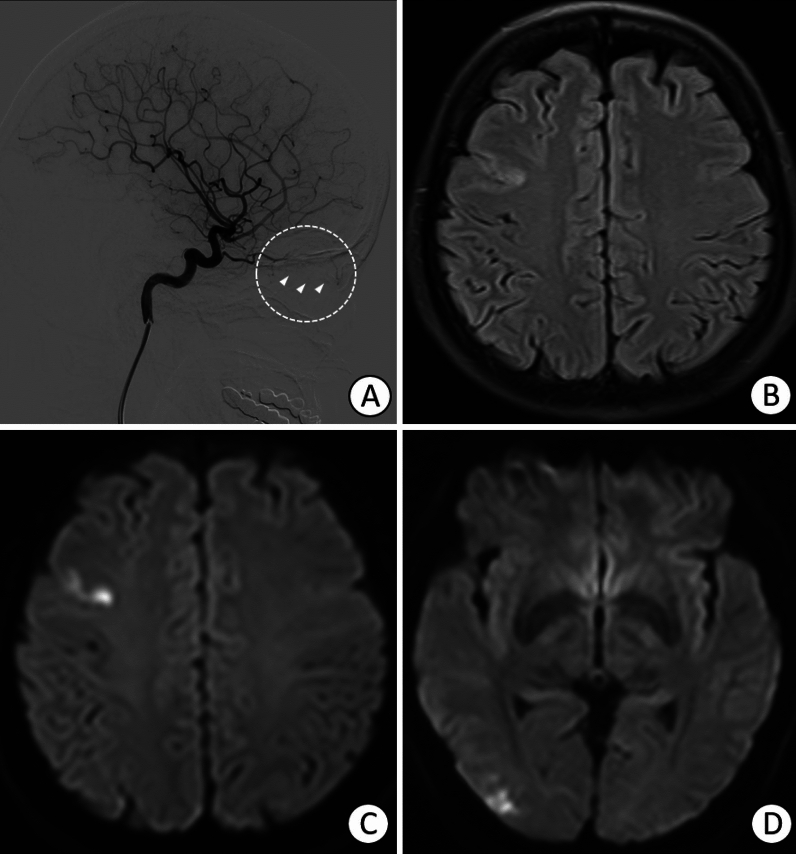
(**A**) Right internal carotid angiogram showing right central retinal artery occlusion, as shown by the circle and arrows. (**B**–**D**) Fluid-attenuated inversion recovery and Diffusion-weighted imaging indicating scattered acute infarctions in the right frontal and occipital lobes.

**Figure 6:**
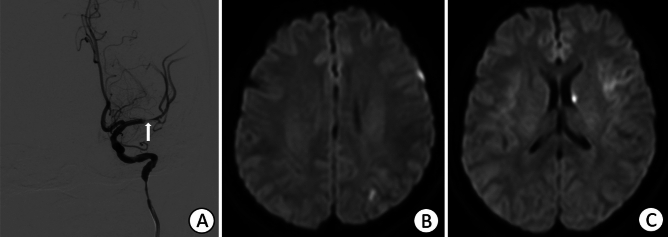
(**A**) The left internal carotid angiogram shows a filling defect in the left middle cerebral artery. (**B**, **C**) Diffusion-weighted images showing several small, acute foci of infarction in the left caudate nucleus and left frontal and parietal lobes.

Catheter angiography: DSA was performed for four patients who received a superselective ophthalmic intra-arterial injection of 1200 U or 1500 U hyaluronidase. Blood flow to the eyeball was compromised, as flow stagnation in the distal branches of the ophthalmic artery was observed in all patients. Patient 1 exhibited right central retinal artery occlusion (Fig. 5) with a small infarction in the right frontal and occipital lobes. A filling defect in the left MCA was observed in Patient 3 (Fig. 6) with multiple cerebral infarction foci.

### Treatment and outcome

The mean hospitalization duration was 6.8 days (range 3–11 days). Four patients received selective ophthalmic intra-arterial administration of hyaluronidase, five patients received retrobulbar hyaluronidase injections, two patients received anterior chamber paracentesis, and one patient received eye massage therapy. Five patients still had no light perception, while two showed significant improvement in visual acuity. Patient 2, who had severe cerebral infarction and postinfarction haemorrhage, was transferred to another hospital. The acute thrombosis lesion of the left MCA in Patient 3 was treated with stent extraction to ensure revascularization. Patients 1, 6 and 10 suffered from acute cerebral infarction and were treated with conservative hyperbaric oxygen therapy (HBOT).

## Discussion

Since the first cases of blindness occurring immediately after aesthetic filler treatments were reported in the 1980s^13^, additional cases of filler injection-induced ophthalmic and cerebral complications have been reported. Only 34 articles describing blindness following aesthetic injectable treatment were found in the PubMed and Medline databases between 2000 and 2022^14^. However, few articles have reported the complete radiological appearance of cerebral and ocular complications. In this study, we summarized the clinical characteristics and radiological findings of twelve patients who received facial filler injections in association with vascular complications. Despite the more commonly involved OAO or its branch in all patients who underwent filler injection, cerebral infarctions on MRI were found in five patients. The radiological findings included filling defects in the MCA and flow stagnation in the distal branches of the ophthalmic artery on DSA, OA occlusion on CTA, optic nerve ischaemia, cerebral infarction on conventional brain MRI and haemorrhages on SWI.

Patients with HA-associated severe vascular complications of the eye and brain mostly present with symptoms such as blurred vision, headache, nausea, and vomiting^15^. The mechanism of vascular complications may be related to the abundance of anastomotic vessels and inappropriate surgical operation^16^. The blood supply in most facial injection areas mainly arises from the ophthalmic artery and internal carotid artery branches. The ophthalmic artery has multiple branches, such as the dorsal nasal artery, angular artery, supratrochlear artery and supraorbital artery, which anastomose with many other arteries in the face^17^. When injections are performed in facial areas, the tip of the needle may penetrate the artery; then, when the plunger pressure exceeds the systolic blood pressure, the filler can reverse the flow in the artery, moving as an embolus that propagates towards the origin of the ophthalmic artery, retinal artery, and internal carotid artery, subsequently reaching the cerebral circulation and causing brain damage^4,13^. The possible entrance of retrograde flow varies with the injection site: The supratrochlear and supraorbital arteries are possible entry sites for retrograde blood flow in the glabellar and forehead regions. Anastomosis of the dorsal nasal artery from the ophthalmic artery, angular artery, or lateral nasal artery with the facial artery is the possible entry point for retrograde flow in the nose and nasolabial fold^18,19^. Occlusion of the ophthalmic artery was mostly reported due to injections in the nose, and six patients had optic nerve ischaemia, which may have been caused by the ophthalmic artery. However, occlusion of the retinal artery was mainly due to injections in the glabella. Three patients in this study received HA injections in the glabellae, and two of them developed clinical complications due to embolization of the retinal arteries, which is in accordance with the mechanism described above.

Two patients in our study who were injected on only one side of the face suffered bilateral cerebral infarction, possibly due to retrograde flow into the circle of Willis, through which the embolus arrived at the contralateral cerebral hemisphere, similar to the findings of a previous study^20^. Autologous fat causes unilateral permanent blindness more frequently than HA does. Compared with autologous fat, HA was reported by Park et al.^9^ to be more likely to obstruct distal branches of the ophthalmic artery. However, unlike their report that large filling defects were only visible in fat-injected patients, in our HA-injected patients, large filling defects were found in the left MCA in Patient 3 and in the right OAO in Patient 2, illustrating that HA-related embolisms can also cause cerebral infarction and vision loss. One patient had a history of breast cancer, and a blood test showed a hypercoagulable state. Her left-eye blindness and intracranial hypertension occurred within 30 min of HA filler injection. We speculate that this may have been due to the complicated pathogenesis of hypercoagulation combined with HA injection. In a previous study^21^, cerebral haemorrhage caused by superior sagittal sinus thrombosis immediately after HA injection was reported, possibly due to the erroneous injection of HA particles into veins during administration; these particles then passed through the venous system to the cavernous sinus due to the frontal vein plexus and the absence of a venous valve in the facial area, ultimately causing blockage in the superior sagittal sinus. All the patients in our study showed intracranial sinus normal, and no sinus thrombosis was observed. Cerebral haemorrhage after cosmetic facial injection has rarely been reported^22^; however, postinfarction haemorrhage formation and SAH were found in this case series, and the pathogenesis of these conditions remains unclear. Even with optimal medical therapy, malignant cerebral infarction is associated with up to 80% mortality in the first week^23^; however, Patient 2, who experienced cerebral haemorrhage after malignant cerebral infarction, was transferred to another hospital, and she was reported to have partly recovered at the 2-month follow-up. Three patients with cerebral infarction in this study underwent HBOT instead of thrombolysis because they were admitted to the hospital at that time for loss of vision and the cerebral infarction was subsequently detected on MRI, however, the time window for thrombolysis had passed. Nguyen NB et al. proposed HBOT is a non-drug treatment that could reduce functional symptoms, improve mobility, and reduce treatment time for patients with cerebral infarction^24^.

According to a meta-analysis, cerebral infarction occurs in 12.9–24% of patients with serious complications after facial injections^25^. In a previous national survey by the Korean Retina Society^10^, patients with cerebral infarction were found among those with occlusion of the ophthalmic artery and its branches following facial filler injections. Among thirty-one patients who underwent brain MR imaging, 12 (39%) had focal or multifocal brain infarctions; however, only six of these patients had concomitant neurologic symptoms, comprising contralateral hemiplegia and dysarthria. In our study, 5 of twelve patients (41.7%) had brain infarctions, and only three of them reported neurologic symptoms. Most patients suffer cerebral infarction during or shortly after surgery, however, five patients with a late onset of 9 h have been reported^26^. According to Xin L et al.^27^, only 15% of patients confirmed with cerebral infarction by MRI or angiography have neurologic symptoms. In a previous study of multiple abnormal cerebral imaging changes in four out of ten patients with emotional disorder syndrome after cosmetic facial injection^28^, the author speculated that the abnormalities on MRI might be attributed to cerebral infarction. In a study by Ansari et al.^20^, one patient who reported no focal neurologic deficits was ultimately identified as having multifocal cerebral infarction on MRI. Similarly, Patients 1 and 6 showed no neurological deficits or symptoms, while MRI showed multifocal acute cerebral infarctions.

Filler-associated cerebral infarction has been more frequently observed in patients who had received autologous fat filler than in those who had received HA filler^14^. In this study, five-sixths of the patients were injected with HA, and nearly half of the patients (five out of twelve) suffered concomitant cerebral infarction, sometimes even bilateral or multifocal, on MRI. Eight CT scans performed for six patients after onset revealed no cerebral infarction or haemorrhage; however, the subsequent MRI showed multifocal acute/subacute cerebral infarctions. To obtain high-resolution imaging in soft tissue and sensitivity in detecting ischaemia, we strongly suggest that MRI should be performed at the earliest convenience to detect early ischaemia for early intervention even if no neurological symptoms are present. However, in this study, four patients with vision loss after filler injection did not undergo MRI scans because they presented no neurotic symptoms. It is believed that the actual incidence of cerebral ischaemia is higher than that reported, given that many HA filling surgeries are performed every year and because of the lack of brain MRI data for non-to-slightly symptomatic patients. It is necessary for patients who have visual loss to undergo an MRI scan for potential cerebral and optic nerve damage^29^.

Although hyaluronidase can effectively degrade hyaluronic acid in the skin, the use of retrobulbar injections of hyaluronidase for reversing HA-related blindness remains controversial^30^. Theoretically, hyaluronidase could be useful for preventing HA-derived cerebral embolisms in the early stages. The appropriate dose of hyaluronidase is considered to be 2–4 mL (1500 U). Superselective intra-arterial thrombolysis has also been recently reported. Xiao et al. proposed that timely IATT is effective for ocular artery embolism caused by facial filler injections^8^. In our study, the endovascular administration of hyaluronidase alleviated occlusion of the ophthalmic artery and its branches in five patients, but only two patients experienced visual improvement after treatment. Thus, superselective angiographic delivery of hyaluronidase may have limited effects on reversing vision. Despite the use of hyaluronidase, the low recovery rate could be partially explained by the excessive gap between symptom onset and hyaluronidase injection, which ranged from 0.5 to 72 h in our study, with nine of the twelve patients exceeding the four-hour threshold. However, all the patients were transferred from other clinics, and a preoperative examination was needed, so it was difficult to manage the patients within the golden hour^31^. Zhang et al.^32^ concluded that the combined use of hyaluronidase and urokinase is more effective than hyaluronidase alone. In-depth knowledge of the complex anatomy of the nose-eye-cerebral vasculature^4^ and the use of a gentle technique for injecting the filler using the right pressure and selection of the proper region with a small volume are crucial factors to avoid serious consequences. Aspiration before injection might prevent retrograde embolization of the filler. Additionally, a blunt needle or cannula is recommended to avoid piercing into blood vessels and probable subsequent complications.

Despite the twelve valuable cases presented herein, this study has several limitations. First, a small population was included, although this sample size was larger than that of many previous case reports that included only one patient. Second, we excluded patients by searching for keywords in the electronic medical records, which may have introduced selection bias. Finally, not all patients underwent brain MR, as CT is not sensitive for detecting small early cerebral infarctions, and patients with brain damage may have been overlooked and underestimated.

In summary, cosmetic filler injections can result in emergent and catastrophic cerebral and ocular complications such as blindness and cerebral infarction. Initial radiological examinations, especially MRI, are crucial for detecting stroke, as some strokes may be asymptomatic. Awareness of severe complications may help both injectors to avoid vascular adverse events and clinicians in the treatment of complications immediately and properly.

## Data Availability

The authors declare that they had full access to all of the data in this study and that the authors take complete responsibility for the integrity of the data and the accuracy of the data analysis. The datasets used or analysed during the current study are available from the first author upon reasonable request.
